# 924. D-Mannose Metabolome Regulates Influenza A Disease via Immunometabolic Reprogramming

**DOI:** 10.1093/ofid/ofad500.969

**Published:** 2023-11-27

**Authors:** Ronghui Liang, Shuofeng Yuan, Ziwei Ye, Jasper F W Chan

**Affiliations:** The University of Hong Kong, HONG KONG, Hong Kong; The University of Hong Kong, HONG KONG, Hong Kong; The University of Hong Kong, HONG KONG, Hong Kong; The University of Hong Kong, HONG KONG, Hong Kong

## Abstract

**Background:**

Host survival depends on the elimination of viruses and mitigation of tissue damage. Host tolerance to viruses depends on the severity of collateral damage by exuberant innate immune response which also diverts energy away from essential physiological functions.

**Methods:**

Using influenza A H1N1 virus as the study object, we performed targeted quantitation of metabolites on the human lung A549 cells to demonstrate the metabolic profile before and after virus infection. Meanwhile, the real-time glycolysis rate with and without D-mannose supplement, in the context of H1N1 infection, was monitored by the Seahorse XF Analyzers. For in vivo study, BALB/c mice (n=5 per group) were orally administrated with 2g/kg/day of mannose or PBS control after lethal challenge with H1N1 until 6 days post-infection (dpi), followed by health surveillance until 14dpi. For the delayed antiviral treatment, we recorded the survival rate of H1N1-infected balb/c mice with the combinational treatment of zanamivir (50mg/kg/day, I.P.) and mannose (2g/kg/day, P.O.) from 2dpi to 6dpi. The mice receiving PBS treatment or zanamivir alone were taken as controls (n=5).

**Results:**

The modulation of D-mannose flux rewired the H1N1-triggered immunometabolic response cascade and reduced tissue damage. Mechanistically, D-mannose competed with glucose with the same transporter(s) and hexokinase but accumulates as mannose-6-phosphate, which slowed down the glycolysis rate, reduced mitochondrial reactive-oxygen-species and succinate-mediated hypoxia-inducible factor-1α, and thus reduced virus-induced proinflammatory cytokine production. Administration of D-mannose alone or in combination with Zanamivir protects against mortality after a lethal dose of influenza A virus challenge in vivo.

**Conclusion:**

These results highlight a potential therapeutic strategy in which reprogramming of host-metabolism by D-mannose for the therapeutic cure of influenza diseases.

**Disclosures:**

**All Authors**: No reported disclosures

